# Krill oil for knee osteoarthritis: A meta-analysis of randomized controlled trials

**DOI:** 10.1097/MD.0000000000041566

**Published:** 2025-02-14

**Authors:** Jiahao Meng, Xuanyu Wang, Yinghui Li, Yuqing Xiang, Yumei Wu, Yilin Xiong, Pan Liu, Shuguang Gao

**Affiliations:** a Department of Orthopaedics, Xiangya Hospital, Central South University, Changsha, Hunan, China; b Key Laboratory of Aging-related Bone and Joint Diseases Prevention and Treatment, Ministry of Education, Xiangya Hospital, Central South University, Changsha, China; c Xiangya School of Medicine, Central South University, Changsha, Hunan, China; d Faculty of law, Central South University of Forestry and Technology, Changsha, Hunan, China; e National Clinical Research Center of Geriatric Disorders, Xiangya Hospital, Central South University, Changsha, Hunan, China.

**Keywords:** knee osteoarthritis, krill oil, meta-analysis of randomized controlled trials, visual analog scale, Western Ontario and McMaster Universities Osteoarthritis Index

## Abstract

**Background:**

Knee osteoarthritis, a prevalent musculoskeletal disorder, significantly impacts global health and quality of life. Unfortunately, there is no disease modifying osteoarthritis drugs until now. Krill oil is being explored as a potential alternative, however its efficacy in managing knee symptoms remains unclear. Therefore, the meta-analysis of krill oil in knee osteoarthritis would be interesting and useful.

**Methods:**

We conducted a systematic search of PubMed, Cochrane Library, Embase, and Web of Science databases from their inception through November 28, 2024, employing predefined search terms, including “krill oil” and “knee osteoarthritis.” We included all relevant randomized controlled trials to ensure a comprehensive analysis. Visual analog scale and Western Ontario and McMaster Universities Osteoarthritis Index (WOMAC) of pain, stiffness and function were served as primary outcomes. Moreover, blood markers and adverse events were also included.

**Results:**

Five randomized controlled trials involving 730 participants were included. Relative to the usual care group, the krill oil group demonstrated no significant improvement in knee osteoarthritis as measured by visual analog scale; however, it exhibited significant benefits in terms of pain (standardized mean difference [SMD] −0.60; 95% confidence interval [CI] −0.99 to −0.21), stiffness (SMD −0.59; 95%CI −1.04 to −0.14), and functional outcomes (SMD −0.68; 95% CI −1.09 to −0.27) based on WOMAC assessments. Analysis of blood markers also revealed no significant effects of krill oil group compared to the usual care group. Moreover, adverse events in the krill oil group and usual care group also showed no statistical difference. The safety profiles were similar between the 2 groups.

**Conclusion:**

Krill oil presents as a promising safe therapeutic option for knee osteoarthritis; however, its efficacy in pain relief requires further investigation.

## 1. Introduction

Osteoarthritis (OA) is a prevalent musculoskeletal condition that poses substantial global health challenges, exacerbated by its increasing prevalence and detrimental effects on quality of life. Characterized primarily by joint pain, stiffness, and reduced mobility, knee OA imposes substantial burdens on healthcare systems and economies.^[1]^ The quest for effective interventions to manage knee OA has intensified due to the limited efficacy of current treatments and the desire for safer, more natural alternatives.^[2]^

Krill oil, a marine-derived supplement rich in omega-3 fatty acids and antioxidants, has emerged as a potential agent for managing knee OA symptoms. Laboratory and animal studies have suggested that krill oil may possess anti-inflammatory properties and contribute to joint health.^[3]^ However, the clinical efficacy of krill oil in the treatment of knee OA has been a subject of considerable debate. While some studies have reported positive effects of krill oil on knee pain and function,^[4]^ others have found insufficient evidence to support its widespread use,^[5,6]^ including a meta-analysis recently.^[6]^ This discordance underscores the need for more randomized controlled trials (RCTs) to clarify the role of krill oil in knee OA management.

This paper includes all RCTs with high-quality evidence.^[4,5,7–9]^ Given the need for updated evidence, we conducted a comprehensive meta-analysis to assess the efficacy and safety of krill oil in managing knee OA. Our study synthesizes current evidence, providing clarity on krill oil’s impact on pain reduction, functional improvement, structural disease modification and blood components as well as its safety profile.

## 2. Materials and methods

This meta-analysis of RCTs was performed following the guidelines outlined in the Preferred Reporting Items for Systematic Reviews and Meta-analysis checklist.^[10]^ The study protocol was registered with Prospective Register of Systematic Reviews (CRD42024551707) ensuring transparency and reproducibility.

### 2.1. Search strategy and selection criteria

Until November 28, 2024, we have conducted a comprehensive literature search across several databases, including PubMed, Cochrane Library, Embase, and Web of Science, using predetermined search terms such as “krill oil” and “knee osteoarthritis” and their associated synonyms and Medical Subject Headings terms. Search strategy in detail is in the Supplementary File 1, Supplemental Digital Content, http://links.lww.com/MD/O392. There was no restriction in language. To ensure a thorough search, we also explored Google Scholar for additional relevant literature. The complete details of the search methodology are outlined in the supplementary material. We also retrieved references for all relevant meta-analyses. After the removal of duplicate publications, 2 independent reviewers conducted a preliminary screening of the identified literature based on a set of predefined inclusion and exclusion criteria. Inclusion criteria for study designs in this review encompass RCTs, assessing the efficacy and safety of krill oil in knee osteoarthritis. Exclusions apply to non-randomized studies, reviews, case reports, studies not focused on the specified intervention and those from which the full text could not be accessed. Disagreements regarding the inclusion of a study were resolved through consultation.

### 2.2. Data extraction

A team of 4 reviewers, operating in 2 separate pairs, undertook the task of compiling information from the selected studies for the meta-analysis, adhering to the established inclusion and exclusion parameters. The extracted data included first author, publication year, region, number of participants, demographic characteristics of participants, treatment methods, visual analog scale (VAS), Western Ontario and McMaster Universities Osteoarthritis Index (WOMAC) scores and blood components including High sensitivity C-reactive protein, HDL-cholesterol, LDL-cholesterol, Triglycerides and total cholesterol.

### 2.3. Quality assessment

The same 4 reviewers, divided into 2 independent pairs, evaluated the risk of bias of the included studies using the Cochrane Collaboration’s risk of Bias Tool.^[11]^ Seven potential domains of bias (random sequence generation, allocation concealment, blinding of participants and personnel, blinding of outcome assessment, incomplete outcome data, selective reporting, and other forms of bias) was evaluated. The assessment of potential bias within each domain was meticulously conducted and resulted in a classification that labeled the risk as a low, unclear, or high risk of bias, based on the evidence at hand. When faced with inconsistencies or differing opinions, the reviewing team engaged in collaborative deliberations to reach a mutual agreement.

### 2.4. Primary and secondary outcomes

Primary outcomes included changes in VAS and WOMAC scores of painfulness, stiffness and function, assessed at baseline and at specific postintervention follow-up intervals. Secondary outcomes were C-reactive protein, HDL-cholesterol, LDL-cholesterol, triglycerides, total cholesterol and adverse events.

### 2.5. Statistically analysis

We presented continuous variables as means with their corresponding standard deviations. Binary variables were shown as event number and total number. The Inverse Variance method combined variables. Results were reported as standardized mean difference (SMD) for continuous outcomes and risk ratios for binary outcomes, both accompanied by 95% confidence intervals (CI). A random-effects model was used due to potential heterogeneity. Sensitivity analysis was conducted using the leave-one-out method. Publication bias was assessed using funnel plots, Egger’s, and Begg’s tests. Statistical analyses were done with Review Manager 5.4.1.

## 3. Results

### 3.1. Study selection and study characteristics

**Figure 1.**
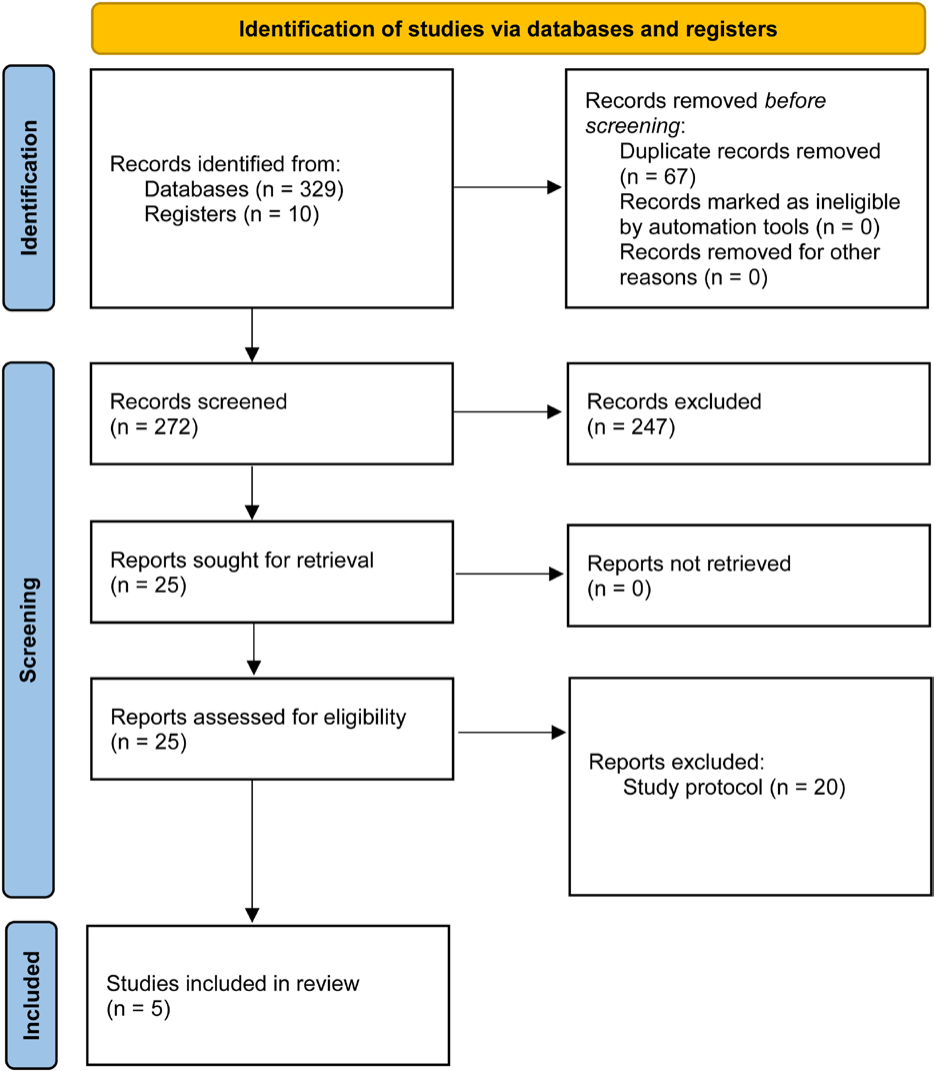
Literature search and selection process.

Figure 1 illustrates the literature search and selection process, which eventually yielded 5 articles.^[4,5,7–9]^ The trials were conducted in various locations, including Australia, Canada, South Korea and Japan, and involved a total of 730 participants. Among the total participants, 347 were allocated to the krill oil intervention, and 240 formed the standard care control group. Interventions involved krill oil dosages varying from 2 to 4 g daily or specific drugs like NKO™ and FP-MD, with treatment duration ranging from 30 days to 24 weeks. Controls in these trials used placebos, such as mixed vegetable oils or identical appearing capsules filled with alternative substances. Two RCTs^[4,9]^ were followed up for 1 month, 1^[7]^ for 12 weeks and 2^[5,8]^ for 24 weeks. Table 1 summarizes the characteristics of the included RCTs.

**Table 1:** Characteristic of included studies.

Study	Location	Patient characteristics	Treatment	
			Intervention	Control
Laslett et al^[5]^	Australia	Patients aged ≥40 yr with ACR criteria for osteoarthritis of the knee, significant knee pain and synovial inflammation	Krill oil (2 g/d) for 24 wk	Placebo for 24 wk
Deutsch^[4]^	Canada	A sum of 90 patients participated with a confirmed diagnosis of cardiovascular disease and/or rheumatoid arthritis and/or osteoarthritis and a C-reactive protein level of more than 1.0 mg/dL for 3 consecutive weeks	300 mg NKO™ for 30 d (17% EPA, 10% DHA and an omega-3 versus omega-6 ratio of 15–1)	Placebo for 30 d (the placebo used was microcrystalline cellulose)
Stonehouse et al^[8]^	Australia	Healthy adults (n = 235, 40–65 yr, BMI > 18.5 to < 35 kg/m²) with a clinical diagnosis of mild-to-moderate knee OA, regular knee pain	4 g krill oil (0.60 g EPA/d, 0.28 g DHA/d, 0.45 mg astaxanthin/d) for 6 mouth	Placebo (mixed vegetable oils) for 6 mouth
Suzuki et al^[9]^	Japan	50 adults (38–85 yr) with mild knee pain	2 g krill oil for 30 d	Placebo for 30 d
Hill et al^[7]^	South Korea	Korean men and women between 30 and 75 yr of age with mild osteoarthritis symptoms	FP-MD (combines 321 mg krill oil, astaxanthin, and lower molecular weight hyaluronic acid) for 12 wk	Placebo once daily for 12 wk

ACR = American College of Radiology, BMI = body mass index, DHA = docosahexaenoic acid, EPA = eicosapentaenoic acid, OA = osteoarthritis, RCTs = randomized controlled trials.

### 3.2. Quality assessment

All studies^[4,5,7–9]^ included in the review were assessed to have a low risk or unknown risk of bias, indicating that the research findings are likely to be reliable and robust. A low risk of bias signifies that the study design, conduct, and reporting were of high quality, reducing the potential for systematic errors that could distort the results. The specific distribution of bias risks, detailed in Figure S1, Supplemental Digital Content, http://links.lww.com/MD/O393.

### 3.3. Primary outcomes

**Figure 2.**
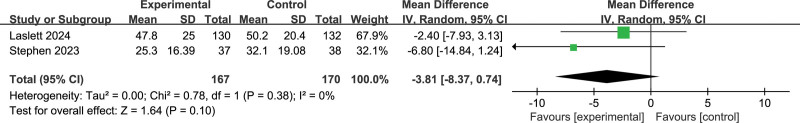
VAS score outcomes. VAS = visual analog scale.

**Figure 3.**
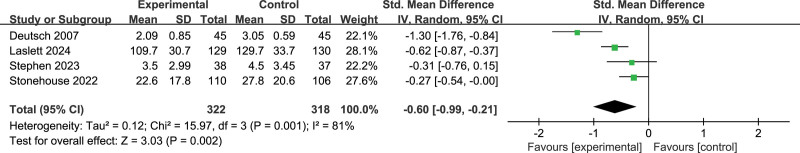
Pain WOMAC score outcomes. WOMAC = Western Ontario and McMaster Universities Osteoarthritis Index.

**Figure 4.**
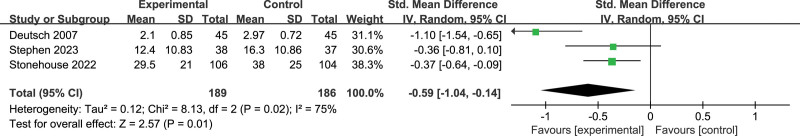
Stiffness WOMAC score outcomes. WOMAC = Western Ontario and McMaster Universities Osteoarthritis Index.

**Figure 5.**
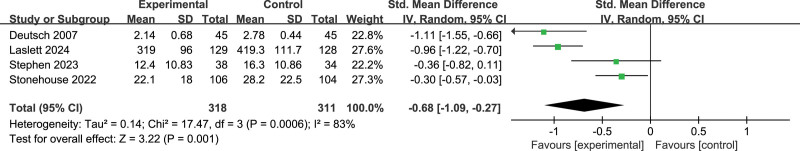
Function WOMAC score outcomes. WOMAC = Western Ontario and McMaster Universities Osteoarthritis Index.

Two RCTs^[5,7]^ reported VAS score outcomes and showed no statistical difference (SMD −3.81; 95% CI −8.37 to 0.74; Fig. 2). Four RCTs^[4,5,7,8]^ reported pain WOMAC score outcomes and showed statistically significant differences (SMD −0.60; 95% CI −0.99 to −0.21; Fig. 3). Three RCTs^[4,7,8]^ reported on stiffness WOMAC score outcomes and showed statistically significant differences (SMD −0.59; 95% CI −1.04 to −0.14; Fig. 4). Four RCTs^[4,5,7,8]^ reported the function WOMAC score outcomes and showed statistically significant differences (SMD −0.68; 95% CI −1.09 to −0.27; Fig. 5).

### 3.4. Secondary outcomes

Four RCTs^[4,5,8,9]^ reported C-reactive protein and showed no statistical difference (SMD −0.62; 95% CI −1.27 to 0.03; Fig. S2, Supplemental Digital Content, http://links.lww.com/MD/O393). Four RCTs^[5,7–9]^ reported on HDL-cholesterol and showed no statistical difference (SMD 0.18; 95% CI −0.20 to 0.55) (Fig. S3, Supplemental Digital Content, http://links.lww.com/MD/O393). Four RCTs^[5,7–9]^ reported on LDL-cholesterol and showed no statistical difference (SMD −0.12; 95% CI −0.54 to 0.30) (Fig. S4, Supplemental Digital Content, http://links.lww.com/MD/O393). Four RCTs^[5,7–9]^ reported on triglycerides and showed no statistical difference (SMD −0.27; 95% CI −0.63 to 0.10; Fig. S5, Supplemental Digital Content, http://links.lww.com/MD/O393). Three RCTs^[7–9]^ reported on total cholesterol and showed no statistical difference (SMD 0.03; 95% CI −0.18 to 0.23; Fig. S6, Supplemental Digital Content, http://links.lww.com/MD/O393). These suggest that the relief of knee osteoarthritis by krill oil may not be related to these substances. Specific mechanisms need to be explored in further studies and RCTs. Besides, 3 RCTs^[4,5,7]^ reported on adverse events which also showed no statistical difference (RR 0.61; 95% CI 0.17–2.11; Fig. S7, Supplemental Digital Content, http://links.lww.com/MD/O393).

### 3.5. Sensitivity analysis

Sensitivity analysis was done using the leave-one-out method on primary outcomes. Excluding any study, the results are robust.

### 3.6. Publication bias

We plotted funnel plots for the main results (WOMAC score) and the results showed symmetry (Figs. S8–S10, Supplemental Digital Content, http://links.lww.com/MD/O393). We performed Egger and Begg statistical tests on all results, which showed *P* > .05 for all results.

## 4. Discussion

Our meta-analysis was conducted to evaluate the efficacy and safety of krill oil in the context of knee OA. The pooled data from the included studies indicate potential modest improvements in knee pain and functionality associated with krill oil supplementation in individuals with knee OA. These findings are in line with the known anti-inflammatory properties of omega-3 fatty acids,^[12]^ key components of krill oil, which are thought to contribute to the alleviation of OA symptoms.^[13]^ The observed benefits of krill oil supplementation could have significant implications for clinical practice, particularly given the limited options for effective, non-pharmacological management of knee OA.^[14]^ Moreover, we found its efficacy in pain relief was less certain by VAS score, but it was statistically effective in pain relief by WOMAC score. This may be due to the inclusion of fewer RCTs and patients in the VAS analysis, resulting in heterogeneity and differences. This needs to be studied in more high quality RCTs.

Importantly, there was a meta-analysis about krill oil supplementation for knee pain on Aug 2024 by Pimentel et al.^[6]^ But we concluded a different conclusion. Pimentel’s study found that krill oil supplementation did not significantly improve knee pain, stiffness, or lipid profile, although it may help knee physical function. Based on these findings, krill oil supplementation is not yet justified for knee pain. However, our research found that krill oil presents potential benefits in reducing stiffness and improving function while its efficacy in pain relief warrants additional investigation. The main reasons are as follows. Firstly, the research objectives are different. Our research is a meta-analysis specifically targeting Krill oil for the treatment of knee osteoarthritis, while Pimentel’s study is for knee joint pain, which may not have accurately included literature related to knee osteoarthritis and krill oil, and there are many confounding factors, making it difficult for clinical reference and application. Secondly, the different literature we included resulted in differences in the results. Pimentel’s study incorporated a Japanese article that was only related to the quality of life associated with mild knee pain.^[15]^ This article does not align with the purpose of our study and is not in English, so we excluded it. Compared to Pimentel’s study, our research includes a high-quality randomized controlled study conducted by Deutsch.^[4]^ The results of this study clearly demonstrate that krill oil significantly suppresses inflammation and alleviates arthritis symptoms within short treatment periods. We believe it is necessary to incorporate this article in our study to make the results more accurate and rigorous.

While the overall trend points towards a positive effect of krill oil, it is crucial to consider the quality of the evidence. The RCTs included in this analysis varied in design, patient demographics, dosage regimens, and outcome measures, which could account for the heterogeneity observed. The methodological quality of the trials was generally high, with most studies employing double-blinding and adequate randomization procedures. However, variations in the duration of treatment and follow-up periods may influence the generalization of the results.

The dosage of krill oil across studies ranged from 2 to 4 g per day, with some trials suggesting a dose-dependent effect on pain reduction and functional improvement. Further investigation is needed to determine the optimal dosage that balances efficacy and safety.^[16]^ It is possible that higher doses may provide greater benefits, but they could also increase the risk of adverse events.

Understanding the precise mechanisms underlying krill oil’s efficacy in knee OA is an area ripe for future research.^[17]^ The rich content of omega-3 fatty acids, particularly eicosapentaenoic acid and docosahexaenoic acid, is believed to modulate inflammatory pathways and reduce the production of pro-inflammatory mediators.^[18]^ Additionally, the antioxidant astaxanthin present in krill oil may contribute to its therapeutic effects by scavenging free radicals and reducing oxidative stress, a known factor in the pathogenesis of OA.^[19]^

Safety data from the included studies indicate that krill oil is generally well-tolerated, with a low incidence of adverse events. However, larger-scale studies with long-term follow-up are necessary to fully understand its safety profile. Of particular concern are the potential interactions between krill oil and medications commonly used by individuals with OA, such as non-steroidal anti-inflammatory drugs.^[8]^

As with any nutritional supplement, it is essential for healthcare providers to counsel patients on the appropriate use of krill oil, potential risks, and the importance of a balanced diet and regular physical activity in managing knee OA. The role of krill oil in the prevention of knee OA and its potential synergies with other treatments warrants exploration to fully harness its therapeutic potential.^[20]^ With a growing elderly population and an increasing burden of OA worldwide,^[21]^ innovative and safe treatment options like krill oil are crucial to improving patient outcomes and reducing the socioeconomic impact of this condition.^[22]^

While our meta-analysis provides valuable insights into the potential efficacy and safety of krill oil for the treatment of knee osteoarthritis, several limitations must be acknowledged. Firstly, the number of RCTs included in this analysis is relatively modest, which may limit the statistical power to detect small but potentially important effects. The heterogeneity across studies in terms of patient demographics, treatment dosages, and duration of intervention further complicates the interpretation of the pooled results. Secondly, the duration of treatment in the included studies was predominantly short to moderate term, with the longest follow-up being 24 weeks. This raises concerns about the sustainability of the observed benefits and the long-term efficacy of krill oil supplementation in managing knee osteoarthritis. Long-term studies are needed to assess whether the benefits of krill oil are maintained over time and whether they can contribute to slowing disease progression. Thirdly, the generalizability of the findings may be limited by the inclusion of predominantly middle-aged and older adults, with limited data available on younger populations or specific subgroups such as those with severe osteoarthritis. Future research should aim to include a more diverse patient population to better understand the applicability of krill oil across different demographic groups. Fourthly, while the methodological quality of the included RCTs was generally high, there were variations in the reporting of outcomes, which could introduce reporting bias. Additionally, the potential for residual confounding factors, such as differences in diet, physical activity levels, and concomitant medications, cannot be ruled out and may influence the observed treatment effects. Lastly, the meta-analysis did not include an assessment of the economic implications of krill oil supplementation, which is an important consideration for healthcare decision-makers. Future research should also consider the cost-effectiveness of krill oil as an adjunct treatment for knee osteoarthritis.

## 5. Conclusion

Krill oil presents as a promising safe therapeutic option for knee osteoarthritis, with potential benefits in reducing stiffness and improving function, however, its efficacy in pain relief warrants additional investigation.

## Acknowledgments

We gratefully acknowledge the help of the Epidemic Statistics Unit Support Team from Hunan Key Laboratory of Joint Degeneration and Injury.

## Author contributions

**Conceptualization:** Jiahao Meng, Yinghui Li, Yumei Wu, Pan Liu, Shuguang Gao.

**Data curation:** Jiahao Meng, Xuanyu Wang.

**Formal analysis:** Jiahao Meng, Xuanyu Wang.

**Methodology:** Jiahao Meng, Xuanyu Wang, Yuqing Xiang, Yinghui Li, Yumei Wu, Pan Liu.

**Project administration:** Jiahao Meng.

**Resources:** Jiahao Meng, Xuanyu Wang.

**Software:** Xuanyu Wang.

**Supervision:** Jiahao Meng, Yuqing Xiang, Yinghui Li, Yumei Wu, Yilin Xiong, Pan Liu, Shuguang Gao.

**Validation:** Jiahao Meng, Xuanyu Wang, Shuguang Gao.

**Visualization:** Jiahao Meng, Xuanyu Wang, Yilin Xiong.

**Writing – original draft:** Xuanyu Wang.

**Writing – review & editing:** Jiahao Meng, Xuanyu Wang, Yuqing Xiang, Yinghui Li, Yumei Wu, Yilin Xiong, Pan Liu, Shuguang Gao.

## Supplementary Material


