# Supplementary material for: Krill oil for knee osteoarthritis: A meta-analysis of randomized controlled trials
Source: Medicine (Baltimore). 2025 Feb 14;104(7):e41566. doi: 10.1097/MD.0000000000041566 (PMC11835064; doi:10.1097/MD.0000000000041566)
Supplement: Supplementary file 1 [file medi-104-e41566-s001.docx]

PubMed(6)

1. ("euphausiacea"[MeSH Terms] OR "euphausiacea"[All Fields] OR "krill"[All Fields]) AND "oil"[All Fields]
2. ("osteoarthritis, knee"[MeSH Terms] OR ("osteoarthritis"[All Fields] AND "knee"[All Fields]) OR "knee osteoarthritis"[All Fields] OR ("knee"[All Fields] AND "osteoarthritis"[All Fields]))
3. ((compar*[tiab]) OR ((singl*[tiab] or doubl*[tiab] or tripl*[tiab]) and (mask*[tiab] or blind*[tiab]))) OR (random*[tiab] or placebo[tiab] or controlled[tiab] or trial*[tiab])
4. #1 And #2 And #3

Cochrane Library (22)

1. MeSH descriptor: [Euphausiacea] explode all trees
2. (Euphausiacea):ti,ab,kw OR (krill):ti,ab,kw (Word variations have been searched)
3. #1 OR #2
4. (oil):ti,ab
5. #3 AND #4
6. MeSH descriptor: [osteoarthritis, knee] explode all trees
7. (osteoarthritis):ti,ab,kw OR (knee):ti,ab,kw OR (knee osteoarthritis):ti,ab,kw (Word variations have been searched)
8. #6 OR #7
9. ((compar*) OR ((singl* or doubl* or tripl*) and (mask* or blind*))) OR (random* or placebo or controlled or trial*):ti,ab
10. #3 AND #5 AND #8

Embase(278)

1. 'krill'/exp OR ‘krill’
2. ‘Euphausiacea’:ab,ti OR ‘krill’:ab,ti
3. #1 OR #2
4. ‘Krill oil’:ab,ti
5. #3 AND #4
6. ‘osteoarthritis'/exp OR 'knee osteoarthritis'
7. ‘osteoarthritis’:ab,ti OR ‘knee’:ab,ti OR ‘knee osteoarthritis’:ab,ti
8. #6 OR #7
9. ((compar*) OR ((singl* or doubl* or tripl*) and (mask* or blind*))) OR (random* or placebo or controlled or trial*):ti,ab
10. #3 AND #5 AND #9

Web of Science (23)

1. TS=(euphausiacea* OR krill* )
2. TS=(oil*)
3. #1 AND #2
4. TS=(osteoarthritis* OR knee osteoarthritis* OR knee*)
5. TS=(((compar*) OR ((singl* or doubl* or tripl*) and (mask* or blind*))) OR (random* or placebo or controlled or trial*))
6. #3 AND #4 AND #5
