# Supplementary material for: Krill oil for knee osteoarthritis: A meta-analysis of randomized controlled trials
Source: Medicine (Baltimore). 2025 Feb 14;104(7):e41566. doi: 10.1097/MD.0000000000041566 (PMC11835064; doi:10.1097/MD.0000000000041566)
Supplement: Supplementary file 2 [file medi-104-e41566-s002.pdf]

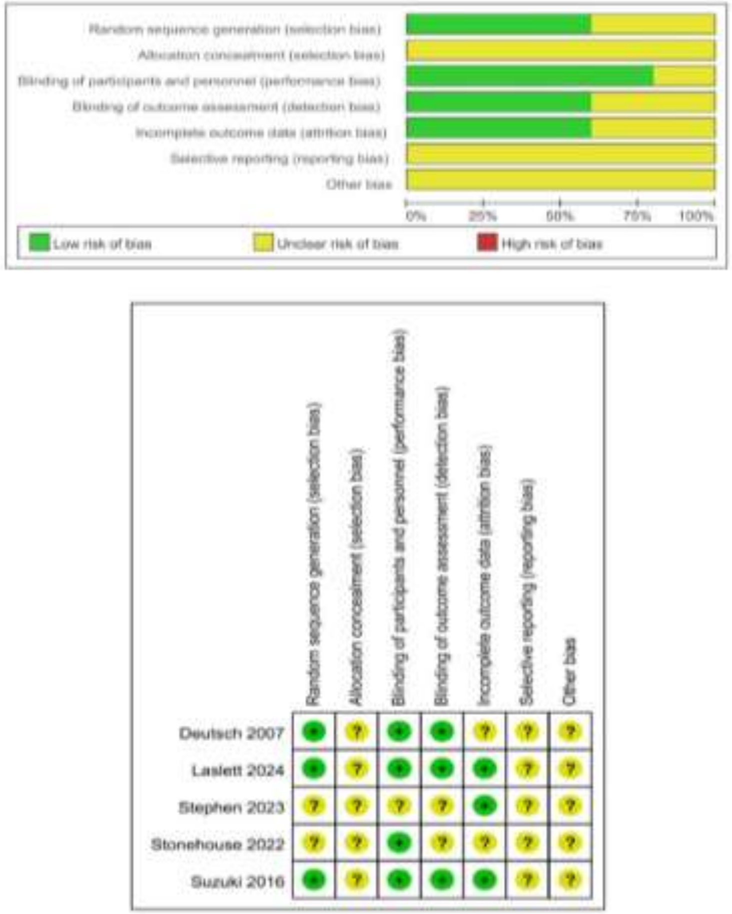

eFigure1. Risk of bias of each included study by Review Manager 5.4.1

	Experimental			Control				Std. Mean Difference	Std. Mean Difference
Study or Subgroup	Mean	SD	Total	Mean	SD	Total	Weight	IV, Random, 95% CI	IV, Random, 95% CI
Deutsch 2007	1.72	1	45	3.59	1.05	45	24.0%	-1.81 [-2.30, -1.31]	
Laslett 2024	1.47	1.12	104	2.1	1.65	103	26.5%	-0.45 [-0.72, -0.17]	
Stonehouse 2022	1.3	1.05	107	1.37	1.13	110	26.6%	-0.06 [-0.33, 0.20]	
Suzuki 2016	0.09	0.1	25	0.17	0.47	22	22.9%	-0.24 [-0.81, 0.34]	
**Total (95% CI)**			**281**			**280**	**100.0%**	**-0.62 [-1.27, 0.03]**	

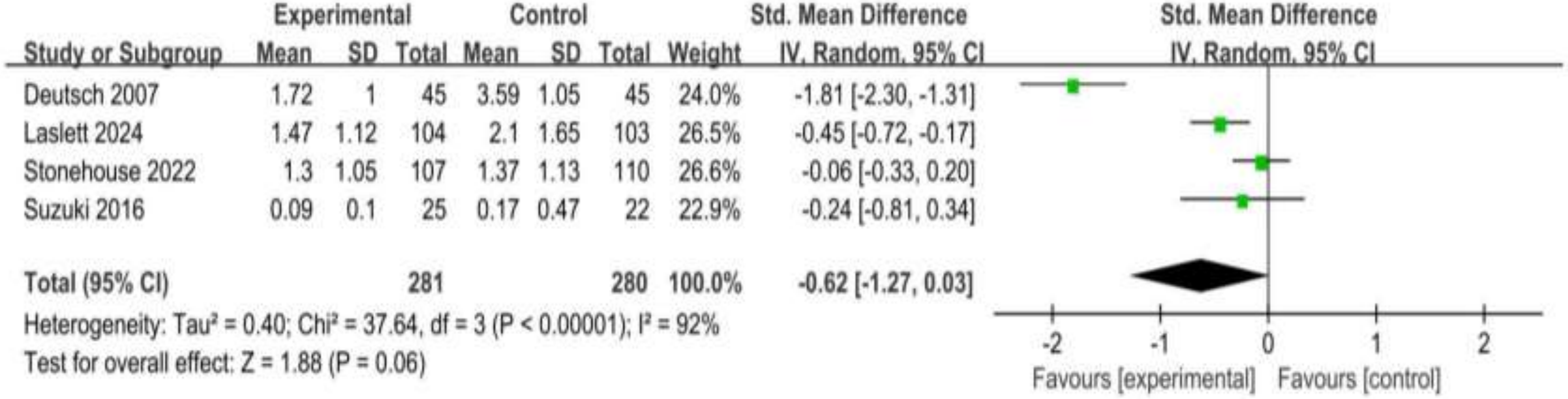

Heterogeneity: Tau² = 0.40; Chi² = 37.64, df = 3 (P < 0.00001); I² = 92%

Test for overall effect: Z = 1.88 (P = 0.06)

eFigure2: krill oil on the C-Reactive Protein

Study or Subgroup	Experimental Mean	Experimental SD	Experimental Total	Control Mean	Control SD	Control Total	Weight	Std. Mean Difference IV, Random, 95% CI
Laslett 2024	1.57	0.07	127	1.5	0.15	127	29.1%	0.60 [0.34, 0.85]
Stephen 2023	56.81	11.42	48	55.78	12.75	45	24.0%	0.08 [-0.32, 0.49]
Stonehouse 2022	1.5	0.4	107	1.4	0.3	103	28.4%	0.28 [0.01, 0.55]
Suzuki 2016	61.6	14.3	25	68.7	12.6	22	18.5%	-0.52 [-1.10, 0.07]
**Total (95% CI)**			**307**			**297**	**100.0%**	**0.18 [-0.20, 0.55]**

Heterogeneity: Tau² = 0.11; Chi² = 13.89, df = 3 (P = 0.003); I² = 78%

Test for overall effect: Z = 0.93 (P = 0.35)

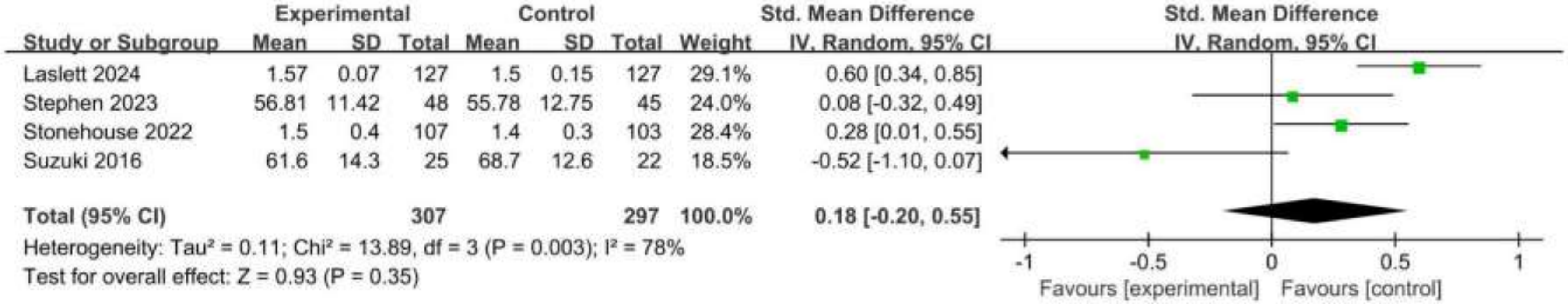

eFigure3: krill oil on the HDL

	Experimental			Control				Std. Mean Difference	Std. Mean Difference
Study or Subgroup	Mean	SD	Total	Mean	SD	Total	Weight	IV, Random, 95% CI	IV, Random, 95% CI
Laslett 2024	3.03	0.22	126	3.2	0.3	127	28.2%	-0.64 [-0.90, -0.39]	
Stephen 2023	111.44	35.61	48	109.91	27.64	45	24.3%	0.05 [-0.36, 0.45]	
Stonehouse 2022	3.8	1.1	107	3.8	1.1	103	27.8%	0.00 [-0.27, 0.27]	
Suzuki 2016	123.5	23.2	25	116.9	30.3	22	19.7%	0.24 [-0.33, 0.82]	
**Total (95% CI)**			**306**			**297**	**100.0%**	**-0.12 [-0.54, 0.30]**	

Heterogeneity: Tau² = 0.14; Chi² = 17.45, df = 3 (P = 0.0006); I² = 83%

Test for overall effect: Z = 0.57 (P = 0.57)

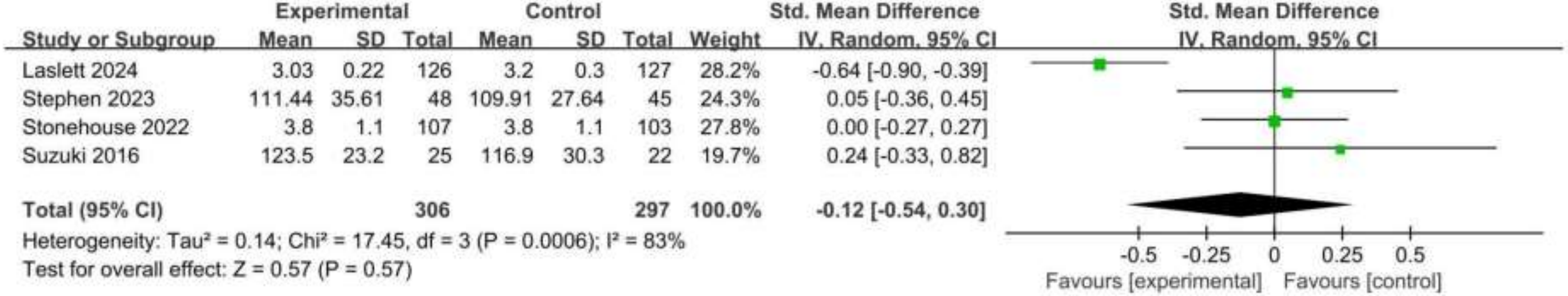

eFigure4: krill oil on the LDL

Study or Subgroup	Experimental Mean	Experimental SD	Experimental Total	Control Mean	Control SD	Control Total	Weight	Std. Mean Difference IV, Random, 95% CI
Laslett 2024	1.3	0.15	128	1.43	0.22	129	29.2%	-0.69 [-0.94, -0.44]
Stephen 2023	126.25	59.17	48	126.02	78.31	45	23.8%	0.00 [-0.40, 0.41]
Stonehouse 2022	1.4	0.8	107	1.5	0.9	103	28.6%	-0.12 [-0.39, 0.15]
Suzuki 2016	118.7	51.3	25	131.5	88.7	22	18.3%	-0.18 [-0.75, 0.40]
**Total (95% CI)**			**308**			**299**	**100.0%**	**-0.27 [-0.63, 0.10]**

Heterogeneity: $Tau^2 = 0.10$; $Chi^2 = 12.99$, df = 3 (P = 0.005); $I^2 = 77\%$

Test for overall effect: Z = 1.44 (P = 0.15)

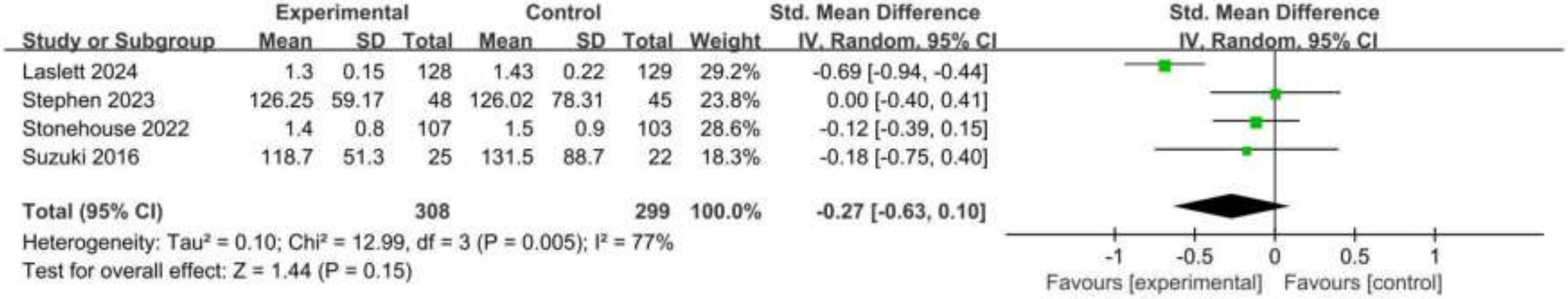

eFigure5: krill oil on the triglycerides

	Experimental			Control				Std. Mean Difference
Study or Subgroup	Mean	SD	Total	Mean	SD	Total	Weight	IV, Random, 95% CI
Stephen 2023	190.69	38.38	48	188.07	26.92	45	26.6%	0.08 [-0.33, 0.48]
Stonehouse 2022	5.9	1.2	107	5.9	1.2	103	60.1%	0.00 [-0.27, 0.27]
Suzuki 2016	212	26.5	25	211	32.5	22	13.4%	0.03 [-0.54, 0.61]
**Total (95% CI)**			**180**			**170**	**100.0%**	**0.03 [-0.18, 0.23]**

Heterogeneity: Tau² = 0.00; Chi² = 0.10, df = 2 (P = 0.95); I² = 0%
Test for overall effect: Z = 0.24 (P = 0.81)

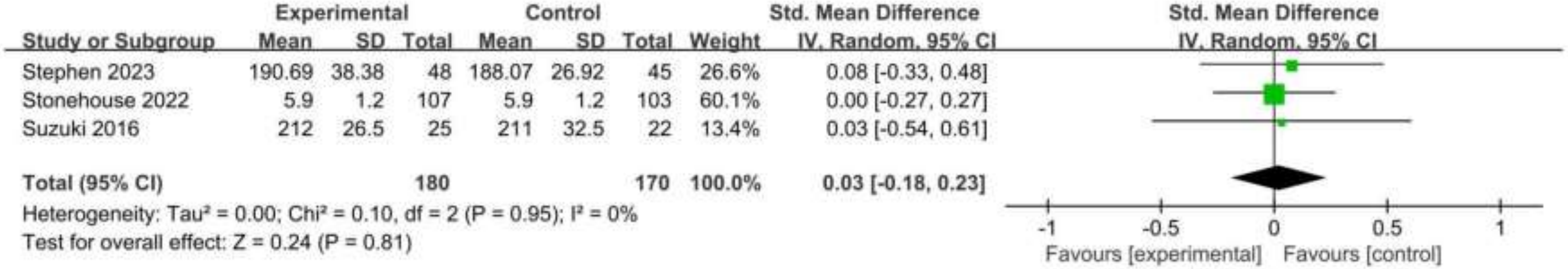

eFigure6: krill oil on the total cholesterol

Study or Subgroup	Experimental Events	Experimental Total	Control Events	Control Total	Weight	Risk Ratio IV, Random, 95% CI
Deutsch 2007	0	45	0	45		Not estimable
Laslett 2024	67	130	71	132	65.8%	0.96 [0.76, 1.21]
Stephen 2023	2	50	8	50	34.2%	0.25 [0.06, 1.12]
**Total (95% CI)**		**225**		**227**	**100.0%**	**0.61 [0.17, 2.11]**
Total events	69		79			

Heterogeneity: $Tau^2 = 0.60$; $Chi^2 = 3.01$, df = 1 (P = 0.08); $I^2 = 67\%$

Test for overall effect: Z = 0.79 (P = 0.43)

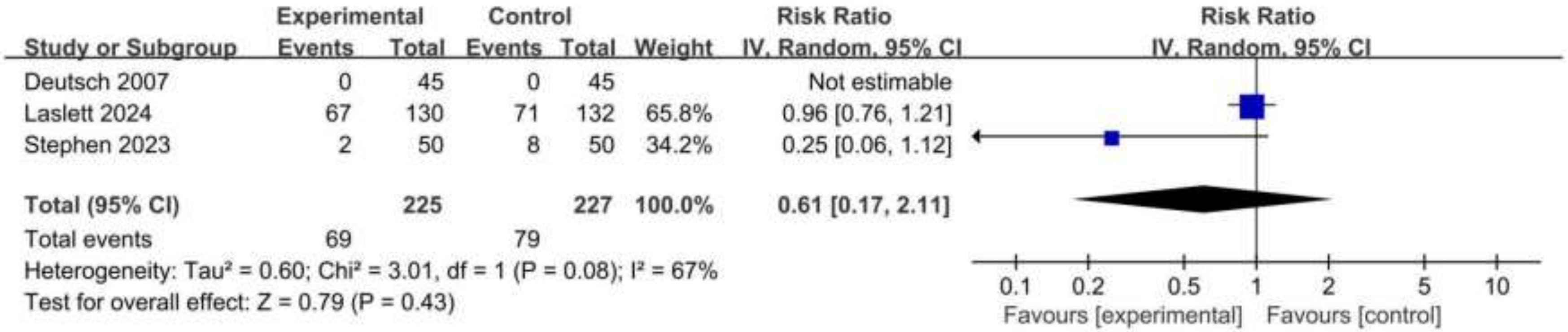

eFigure7: krill oil on the adverse events

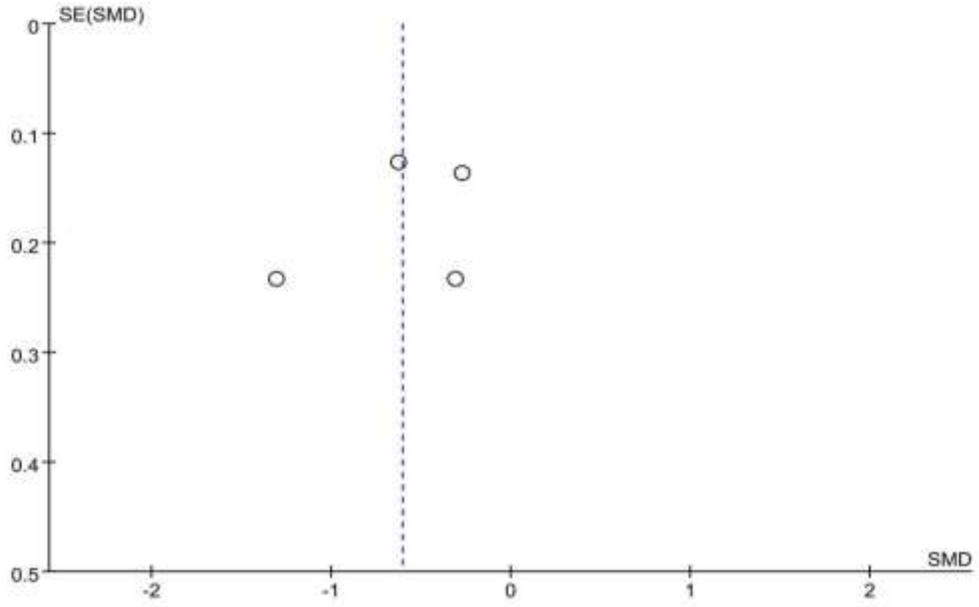

eFigure8: funnel plots for the Pain WOMAC

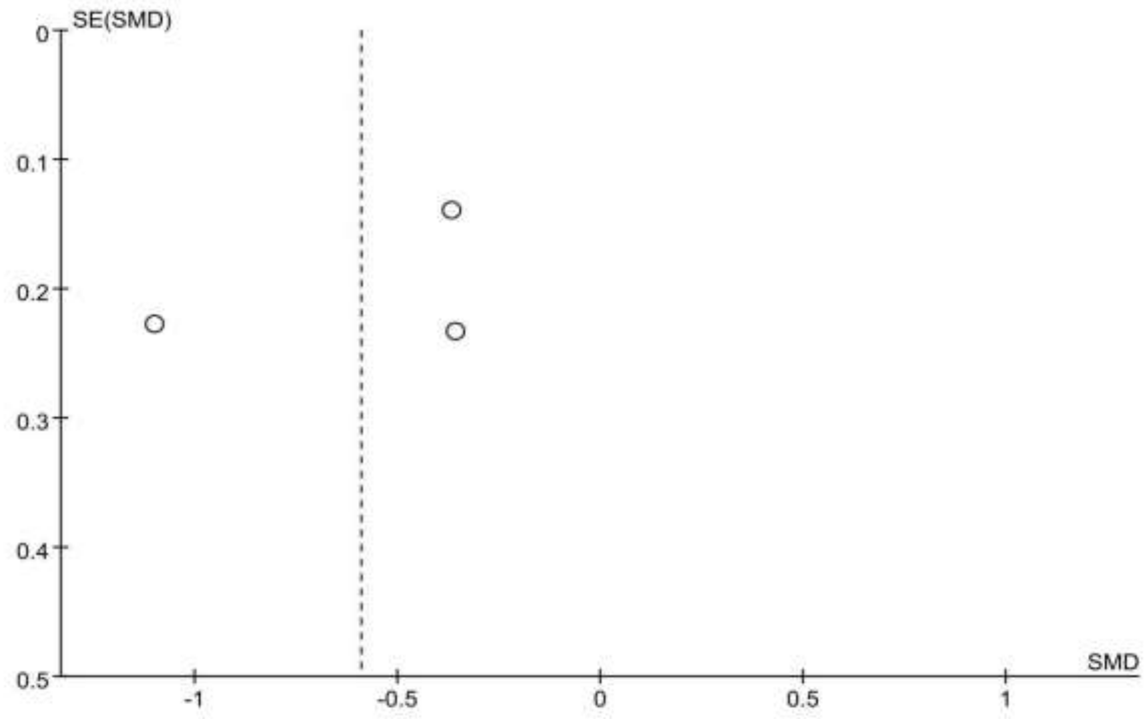

eFigure9: funnel plots for the Stiffness WOMAC

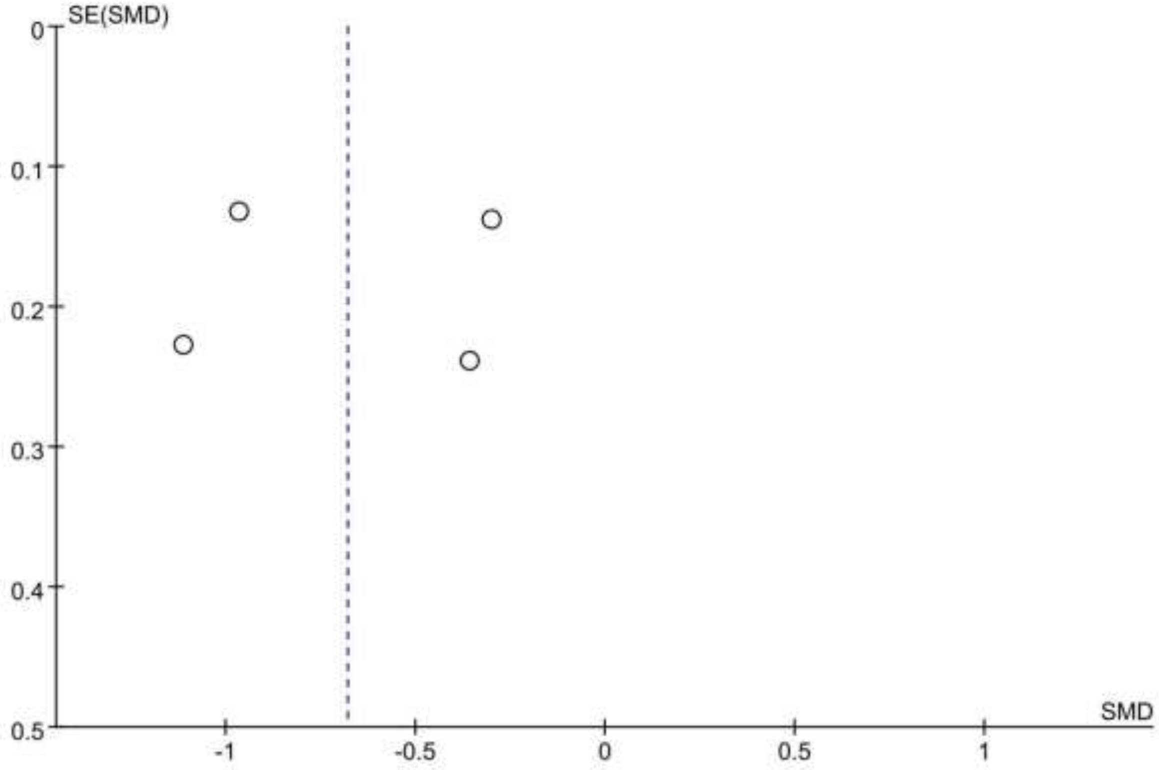

eFigure10: funnel plots for the Functional WOMAC
